# Encouraging communication and cooperation in e-learning: solving and creating new interdisciplinary case histories

**DOI:** 10.3205/zma001458

**Published:** 2021-03-15

**Authors:** Sebastian Ertl, Dagmar Steinmair, Henriette Löffler-Stastka

**Affiliations:** 1 Med. Universität Wien, Universitätsklinik für Psychoanalyse und Psychotherapie, Vienna, Austria; 2 Med. Universität Wien, Teaching Center/Postgraduate Unit, Vienna, Austria

**Keywords:** e-learning, case-based blended learning, distant learning, communication training

## Abstract

**Background: **An increasing number of patients to treat combined with a rapidly growing amount of knowledge to integrate, is challenging for future doctors. To take the medical history and diagnose effectively, to send the patient to an expert, to create a relevant expert to expert communication, to discuss with the patient, the time needed for a decision, should be as short as possible. Investigating medical students’ cognitive processes while solving a patient’s case leads to the conclusion that educators should help and facilitate these reasoning and communication processes. Developments in information technology offer a large variety of tools for educators.

**Method: **Practicing repeated memory retrieval in clinically relevant virtual settings leads to more durable storage of theoretical knowledge, especially when applying the theoretical knowledge to meaningful cases. The cases in the present e-learning tool are only solvable when knowledge is coherent, communicated and well-organized, as they demand combination of different interdisciplinary knowledge-fields. Thus, by practicing in the virtual environment, prospective memory (i.e. the ability to remember to do something in the future) is changed and the intention and attention in learning processes is shaped and adapted to the core requirements of clinical practice.

**Conclusion:** Case-based learning can be a promising approach to teach students how to investigate and ask for important information. This paper focuses on undergraduate education and provides an outlook on possible concepts that can be used in different health care sectors.

## Zusammenfassung

**Hintergrund: **Die immer größer werdende Anzahl an Patienten, sowie eine ständige Zunahme des medizinischen Wissens, das integriert werden soll, stellt für angehende Ärzte eine große Herausforderung dar. Die Zeit, die für eine Anamnese und Diagnosestellung, für eine korrekte Überweisung, für das Expertengespräch untereinander, für den Diskussionsprozess mit dem Patienten, aufgewendet werden muss, sollte so kurz wie möglich gehalten werden. Untersuchungen der kognitiven Prozesse von Medizinstudenten*innen, die sich einen Patientenfall angesehen haben, zeigten, dass der Kommunikations- und Entscheidungsprozess bzw. die Entscheidungsfindung im klinischen Alltag besser gelehrt werden sollte. Entwicklungen neuer Technologien für die Ausbildung bieten eine Vielzahl von Möglichkeiten für die medizinische Lehre.

**Methode:** In einer virtuellen Lernumgebung wird anhand von Fallbeispielen theoretisches Wissen angewandt.

**Fazit:** Fallbasiertes Lernen kann ein Ansatz sein, um den Studierenden zu vermitteln, wie sie einen Patientenfall adäquat bearbeiten und die wesentlichen Informationen abfragen und systemrelevant kommunizieren. Die Arbeit gibt einen Einblick in die Medizinausbildung und kann für Weiterbildung in anderen Gesundheitsberufen verwendet werden.

## Introduction

Whether the teaching and learning contents will be remembered or the forgetting curve will be steep, is already given while learning. In clinical situations, successful and prompt memory recovery is a cue, despite a stressful environment. Communication processes are also concerned. Previous research showed that environmental features and internal cognitive and affective shapes during encoding matter. Accessibility of memory content is better when encoding and retrieval state match [1]. However, erosion of knowledge as well as the need to update it is a fact- requiring a motivation for the continuation of professional development and subsequently the maintenance of competence [2].

By creating learning communities, virtual (or real-life) ones, learners get connected and while expert knowledge can be applied, also social and communication skills can be trained and a connected organization aiming at cooperation, having shared goals is simulated. 

The motivation for learning is essential as it determines the amount of time and energy one is willing to invest for goal achievement in the long-run. Motivation is often unconscious and can be predominantly intrinsic (i.e. autotelic- the purpose lies in and not apart from itself) or extrinsic (i.e. enhanced by reward and attenuated by punishment) [2]. After years of studying and passing exams at the university, students have proven their motivation to learn and deepen their knowledge, but preserving this attitude through the professional life might be challenging [2]. Miller et al. described being responsible for patients as an excellent motivation for active learning [2]. 

Interdisciplinary teaching and learning in medicine requires integration and application of knowledge, making connections between different knowledge fields, thus anticipating the real-life scenario already while learning. Clinical reasoning relies on the ability of performing clinical assessments as much as on expert knowledge and practical experience. 

Communications processes between experts, referrals, communication scenarios in expert-discussions as well as communication to and with patients and their relatives or even to stakeholders and insurance companies for reasons of financing the appropriate care are essential for a modern clinical-reasoning- and clinical-decision-making-process to provide the best point of care with public/patient involvement. Such complex situations have to be trained.

### Education theory 

Regarding education personal factors (e.g. individual goals, values, knowledge gathered over the years) matter as much as environmental factors (e.g. social environment, peer-group) and behavioral factors (learning activities, learning process) [3]. The constitutional and the ability measures and instructional preferences are the most highlighted facts postulated by Coffield et al. in his systematic review of learning styles impacting on student’s learning [4].

*Constitutional measures* like personality traits determining learning success are quite stable over time. Different learning types, regarding the preferred sensory modality (e.g. auditory, visual or kinesthetic) for presentation of new content, have been described, although findings were somewhat conflicting [3], learning material presented in more than one modality, may help [5], [6]. 

*Ability measures* consider that learners have a certain extent of abilities to direct their learning preferences (like focusing-scanning or convergent-divergent learning) [4].

*Instructional preferences* refer to the fact that students tend to have individual preferences and their self – constructed approach to learning. 

Students should get the opportunity to experience learning in-depth (i.e. following curiosity, integrating ideas), rather than just aiming at fulfilling assessment requirements (i.e. surface learning). Students who are capable of combining these two aspects are called strategic learners (i.e. adapt time/steady effort to achieve maximum effect). Moreover, good learners can adapt their learning approach to new courses and learning material [7]. As expected, a positive correlation with final marks and clinical performance and strategic learning have been published [8] – communication processes and their training included. 

Knowles [9] postulated that self – directing is an essential aspect for the inner drive or intrinsic motivation for adults. However, in the learning process, the pacing of learning affected by deadlines or other work obligations is essential, as various competing tasks have to be mastered in limited time. Performance seems to depend on whether the learning task is considered as meaningful enough or whether it matches the field of interest. 

Life-long learning demands independency in learning. However, only when self-awareness about skills and compensation abilities is encouraged, the student’s autonomy is enhanced and personalized learning will be effective without neglecting self-care.

#### E-Learning – the approach for everyone?

E-learning often is claimed as the ideal approach adaptable to every learning style- but unfortunately, not each e-learning service is presented in different styles. The idea of different teaching styles came up when teachers noticed that in order to provide effective learning environments, the individual students’ strengths and weaknesses must be assessed and addressed, aiming for a personalized learning environment. While e-learning might be cost-effective and convenient, and virtual learning environments can be customized and used autonomously, they are often regarded as less personal and interactive. Additionally, it might be challenging to guarantee the authenticity of a student’s work, and security issues are not to be neglected. 

However, the numerous advantages of e-learning make it an interesting addition to teaching as usual. In our view they should be seen as an add-on tool and not as a substitute for conventional learning environments. There is an advantage also for fostering and strengthening clear and structured communication processes. 

#### Blended learning – an interactive approach

E-learning – Technology is a tool, not a teacher. By creating a communication between instructor and learner an impersonal technology centered approach could be avoided. Around 20 years ago, blended learning (BL) was nominated by the American Society for Training and Development as one of the top educational trends [10], [11], [12] and was promoted as the modern way of e-learning providing learning through a combination of traditional and e learning methods. BL, a blend of virtual and face-to-face learning setting, increases possibilities of interaction and it has already become the new routine setting during COVID-19 pandemic. In 2003, Guild published a report on the implementation of BL in corporate training, assuming that 85.2% were using this approach in any way [13]. Only three years later, 93% of all asked institutions have already implemented BL in some way and 60% were using blended learning in one-fifth of their courses [13].

When creating BL environments, both clear concepts and teaching expertise are necessary. The fashion e-learning is applied, and especially the amount of blend between face-to-face and online learning time, varies according to the view of the educators in charge [14], [15], [16], [17]. 

Having a face-to-face class at the beginning can facilitate discussion and critical debate in the e-learning setting, result in a true and supportive “community of inquiry” [18]. Independent of the type of teaching approach, every community of inquiry consists of the same three elements, such as cognitive (i.e. a reflective inquiry including a triggering event, exploration, integration and solution), social (i.e. participants identifying with the community, communicating purposefully, developing relationships), and teaching presence [19]. Moreover, verbal communication is often described as synchronous, whereas written communication as a form of asynchronous learning; the challenge of combining them in the e-learning environment can be demanding. According to Garrison and Anderson the kind of interactions taking place in asynchronous e-learning environments in higher education, lead to this special kind of “distant presence”, even if students worked independently from each other and the interaction relies on text-based group discussions [18]. Usually, a significant level of social and cognitive presence in each communication medium is needed to achieve a high learning level in a community.

**Figure 1:**
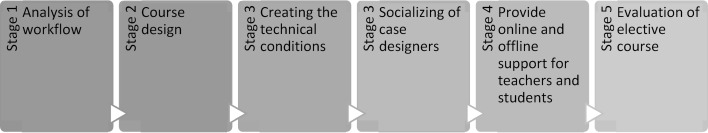
Delivering a case-blended learning course – work flow. ©Ertl 2020, adapted from: A framework for designing and delivering blended e-learning [2], [3], [27], [48].

Implementation of e-learning in current curricula needs effort and investment seeing technology as a curriculum design issue; impact of system-usage on the general education system depends on more than just its availability and usability [3]. Summarizing and reconsidering the aspects as mentioned above we installed a framework for delivering a case-based blended e-learning course (see figure 1 (Fig. 1)).

The aim of the present project was to provide e-learning strategies for students as add on to the existing curriculum at their university. In solving, contributing and reviewing case histories students from different knowledge fields and levels become part of an interacting community of learners. By being a user of the case-history pool as much as a creator and critical reviewer, students earn the fruits of collaborative and team-work as much as they might experience self-efficacy. This model trains them also for further real-life scenarios, as they have to communicate clinical reasoning processes and diagnostic procedures to colleagues via peer-to-peer-feedback, suggest referrals, discuss clinical decisions with peers, explain to the expert, add the consultant’s opinion and reconsider and recall the argumentation towards patients and their relatives. The e-learning platform relies on asynchronous communication, i.e. there is a time-lag between the written inputs and the answer of another participant. Nevertheless, a community of learners is interacting- and as the platform is open to all levels (undergraduate, graduate, post-graduate, clinicians) of all related knowledge-fields, the discussion evolves. Interactive questions are added to give directions especially for younger students, every answer is well-founded and science-based.

## Methods

### Case-based – learning

As the most challenging part for medical students is to handle the huge amount of clinical information, case-based learning could be a promising approach to teach students how to investigate for the important information. Investigating the cognitive actions of medical students solving a patient’s case [20], [21], [22], lead to the conclusion that educators should focus on a higher level of reasoning. In our elective course, we used the teaching concept of case-based blended learning [21], [22], [23]. A significant impact on students’ satisfaction and skills development can be achieved by using a case-based setting instead of traditional teaching methods as Bösner et al. stated [24].

#### Case structure

**Figure 2:**
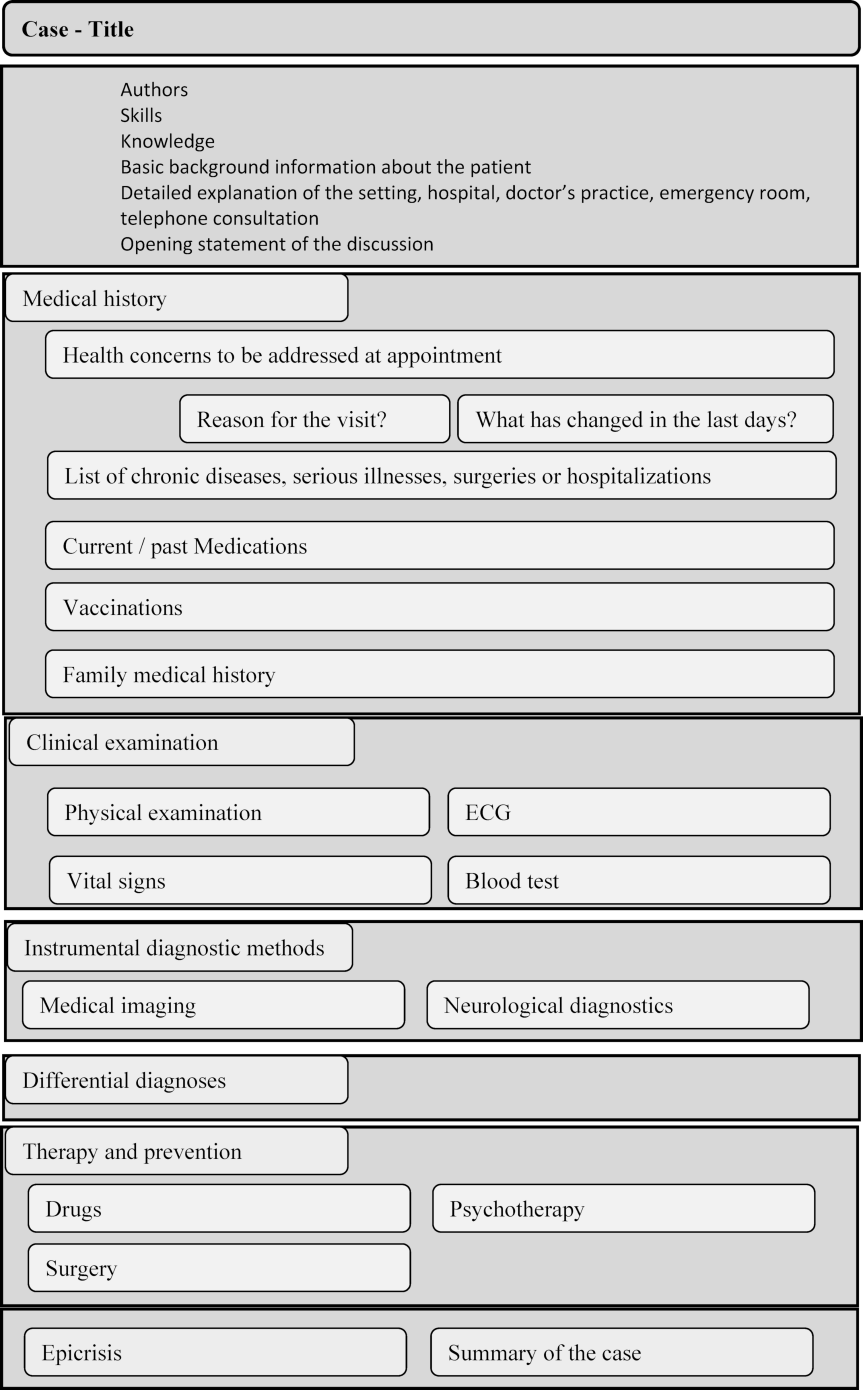
Structure of cases [27]

All cases in our course have the same basic layout [21] to ensure consistently high quality [22], [25] (see figure 2 (Fig. 2)). Each case is designed as a scenario, to simulate a virtual patient, being treated in a hospital. In the beginning, the exact skill and knowledge levels, regarding the current undergraduate curriculum of the Medical University of Vienna, are declared [26]. Thus, students can monitor their growing knowledge by themselves [27]. 

The setting, for instance, a daily ward round or the initial examination in an outpatient clinic, is described in the introduction. The individual items and subgroups are listed in the following figure 2 (Fig. 2).

Examination and test areas are implemented to repeat the lessons learned and deepen the knowledge. 

Web-based learning allows the students to understand the topic by looking up supplemental information and training themselves when they have time. Additionally, because of the detailed information about the setting in which the case history takes place, they can simulate the real life situation using their imagination and adopt the role of the attending physician assessing the medical history, performing the examination and conduct the investigation by coordinating the diagnostic and therapeutic process. Each case starts with the patient’s primary statement and subjectively presented illness complaint to stimulate the learners’ communication skills.

As the e-learning curriculum is an add-on tool completing the medical curriculum at the university of Vienna, the cases match the various modules of the curriculum, enhancing discussion of case histories among students and with their mentors is encouraged. 

There are no knowledge tests but the level of the case history is given and complementary to the curriculum at the medical university of Vienna. The students can achieve the correct results to the case histories directly through the platform. None of the tests implemented are mandatory for the official curriculum and test results of this voluntary curriculum don’t have consequences on grading. Review of case histories takes place continuously through peer-review. As the curriculum is voluntary, users can let curiosity guide their learning process, the “learning workout” is supposed to facilitate enquiry learning.

#### Elective course 

The e-learning takes place in two subsequent steps: The first part of the course consists in the students solving patient cases provided by the e-learning system. In the second part, students are supposed to switch the role, become a teacher, and create a case history for other students. The latter are later rated anonymously by other participants of the elective course in a peer-reviewed setting. This step in our course aims to provide a different perspective, to show our students that teaching and learning needs the effort of both, teachers and students [3], [28].

Once case histories have passed the peer-reviewing, they are submitted to the coordinator for quality check and integration of didactic principles like curriculum fit, content design.

The elective course is offered to all medical students after the second academic year at Medical University Vienna and all students of the University of Vienna attending psychology, chemistry, biochemistry, biology, pharmacy, nutritional sciences, or other similar science subjects. Hence, the ratings and the case histories created by the students with different theoretical backgrounds in the second step of the procedure lead to interdisciplinary communication and exchange. Each academic year, between 80-100 students (17% of the entire cohort) take part in our elective course.

The thematic modules addressed in the e-learning program are shown in table 1 (Tab. 1) [21], [22]. Each module has to be completed successfully and consists of various case histories from different medical disciplines and other specific tasks.

#### Structure of the elective course

Table 1 (Tab. 1) gives an overview of the different thematic modules provided by the e-learning system. Learners can start independently of their learning history and of their academic progress. However, it is synchronized with the academic calendar of the actual curriculum elements provided by the Medical Curriculum Vienna.

## Results

Since the release of our elective course in 2016, 328 students fully completed our online course till May 2020, and 327 answered a detailed questionnaire to measure the support of e-Learning, the attitude of medical students towards eLearning and new learning concepts [29]. The core design has not been changed since the beginning, in order to offer the same condition to every participant; only textual errors or new guidelines have been updated [27], [22], [25]. Some preliminary results of this ongoing study that have shown a significant impact on students grades are stated in [27], [21], [22], [30], [31]: The 4^th^ year ‘s OSCE exam grades (including two communication skills scenarios with simulated patients) got better [21], the learning motivation was effectively triggered [29], [32], collaborative learning was stimulated [25], further clinical research questions [22], didactic considerations [33] and research strategies [34] were brought up, medical history taking was fostered [32] test, or questioning strategies were reformulated [35], extensions to several other medical domains and disciplines have been started [17], and the combination and connection to the face-to-face seminars according to the blended learning approach have been implemented [36].

Communication and collaboration training were implemented by formulating a special request, such as a psychiatric or dermatology consultation, to pass a specific section of a case. These interactive sections are open-ended or multiple-choice questions form, depending on the point of view of the requested department. By putting themselves in the position of the person receiving the explanation, students have to re-think their arguments. 

Students of the elective course reported that their interdisciplinary thinking increased with this step (average rating 0.94±0.888) [27].

It has to be stressed out that this feedback is the participants’ subjective perception; however, the impact on students’ exam grades underlined these results.

So far 140 case histories passed the peer-review and quality check and are already available for training purposes. 

## Discussion

We emphasize that a case-based eLearning tool is an excellent approach for skills and communication training [37], [38], [39] and an ideal setting for OSCE training due to the impact on student’s grades [17], [21]. Case-based learning could be a promising approach when aiming at enhancing medical students’ communication skills. By creating an environment very similar to the clinical one, students adapt to clinical thinking and decision making [40], [41], [42]. Diagnostic reasoning can be seen as case-based problem-solving [43], [44], [45]. According to Kopp et al. [46], learning with “worked examples” or real-world data, leads to acquisition of diagnostic knowledge, especially when erroneous examples with elaborated feedback are provided.

 Case-based learning relies on the findings of previous studies supporting that teaching abstract information does not lead to the same investment of cognitive resources, knowledge integration (e.g. via building up schemas) is not induced to the same extent [46], [47]. Errors in the process of diagnosing can be ascribed to inadequate knowledge, faulty data gathering, and difficulties in clinical reasoning or in the verification process [46]. In the learning environment pointing these errors out, leads to desirable pressure for skill- and knowledge learning, without the severe consequences the same errors would have in a real clinical environment.

Verified feedback entices students on working significantly longer with the offered learning environment. It can be expected that specific diagnostic conclusions can induce deep conceptual understanding [46]. 

Creating case histories and getting feedback via peer-review might be an encouraging learning approach for curious learners and might enhance engram building, this again might be key to the accessibility of the memory content.

The e-learning platform is accessible also to students from other but related knowledge fields and for graduates, postgraduates and clinicians- they all are invited to participate in the peer-review process and answer the open questions arising from the case histories or by providing supplemental material from their expertise fitting the case. Being inclusive also needs consideration, reflexion and stimulates the creation of teaching formats concerning system relevant communication strategies and their training, as it was e.g., essential for the specification of the 4^th^ year’s OSCE [21]. 

As the participation in the platform is truly voluntary also the selection of cases for providing peer-review is free. However, postgraduates get credits for their habilitation curriculum for every peer-review and students from related research fields or nonclinical fields gain clinical insight and gather experience that might prepare them for their professional career and working life. Pharmaceuties’ students for example already use the present e-learning tool for their preparation for their clinical practicum and practical exam. However, experts from all relevant knowledge fields should be even more encouraged to participate in the building up and quality control of the case history library (e.g. by implementing an accreditation system).

### Future directions

The potential for interactivity of our platform is not yet exploited and future research should focus on optimizing the creation of a “community of learners” with emphasis on an all-level approach, including under-, graduates and postgraduate learners from different knowledge fields. Collaborative projects, communication issues and discussion of key concepts between users with different backgrounds could possibly be facilitated by adding user forums, discussion boards and chats concerning specific fields of interest. Thus, future implementations of the evaluation of the platform should focus on assessing users’ expectation, motivation and communication.

## Profiles

**Name of the location:** Medical University of Vienna

**Subject/professional group: **Elective course to support students at the mandatory communication training with standardized patients (SP), offered to all students who have been admitted and registered for a degree study at another Austrian university; co-registration is needed

**Number of learners per year or semester: **2 (professor and tutor), 640 students per year.

**Has a longitudinal communication curriculum been implemented?** Mandatory patient roleplay (PR) with professional actors, trained to act as patients (SP). Different clinical scenarios are offered and trained.

**In which semesters are communication and social skills taught?** In the 2^nd^, 3^rd^ and 4^th^ year.

**What teaching formats are used? **Starting with training for basic medical history starting in the third semester; at the end of the 8th semester, the students have to deal with psychiatric disorders like depression, suicidal tendencies, somatoform disorder, anxiety, and borderline disorder. Always SP used.

**In which semesters are communication and social skills tested? **Courses with continuous assessment for students’ performance, as well as an OSCE at the end of the 8^th^ semester.

**Which examination formats are used? **Courses with continuous assessment for students’ performance, as well as an OSCE at the end of the 8^th^ semester.

**Who is responsible for the development and implementation? **Department of Psychoanalysis and Psychotherapy, Medical University Vienna, Teaching Center, Medical University Vienna.

## Current professional roles of the authors

- Sebastian Ertl: Final year medical student at the Medical University of Vienna. Diploma student and research assistant to Univ. Prof. Dr.in Henriette Löffler-Stastka since 2015. First congress contributions and papers on the subject of case-based learning at international conferences as well as local events. Tutor and co-developer of the elective course: Case-based blended learning.
- Dagmar Steinmair: Doctoral student (clinical neurosciences, CLINS), research assistant at the Medical University of Vienna, Ophthalmologist at UK St. Pölten and Karl Landsteiner University of Health Sciences
- Henriette Löffler-Stastka: Univ. Prof. at Medical University Vienna, Psychiatrist and Psychoanalyst, Dean of Postgraduate Programs.

## Competing interests

The authors declare that they have no competing interests. 

## Figures and Tables

**Table 1:**
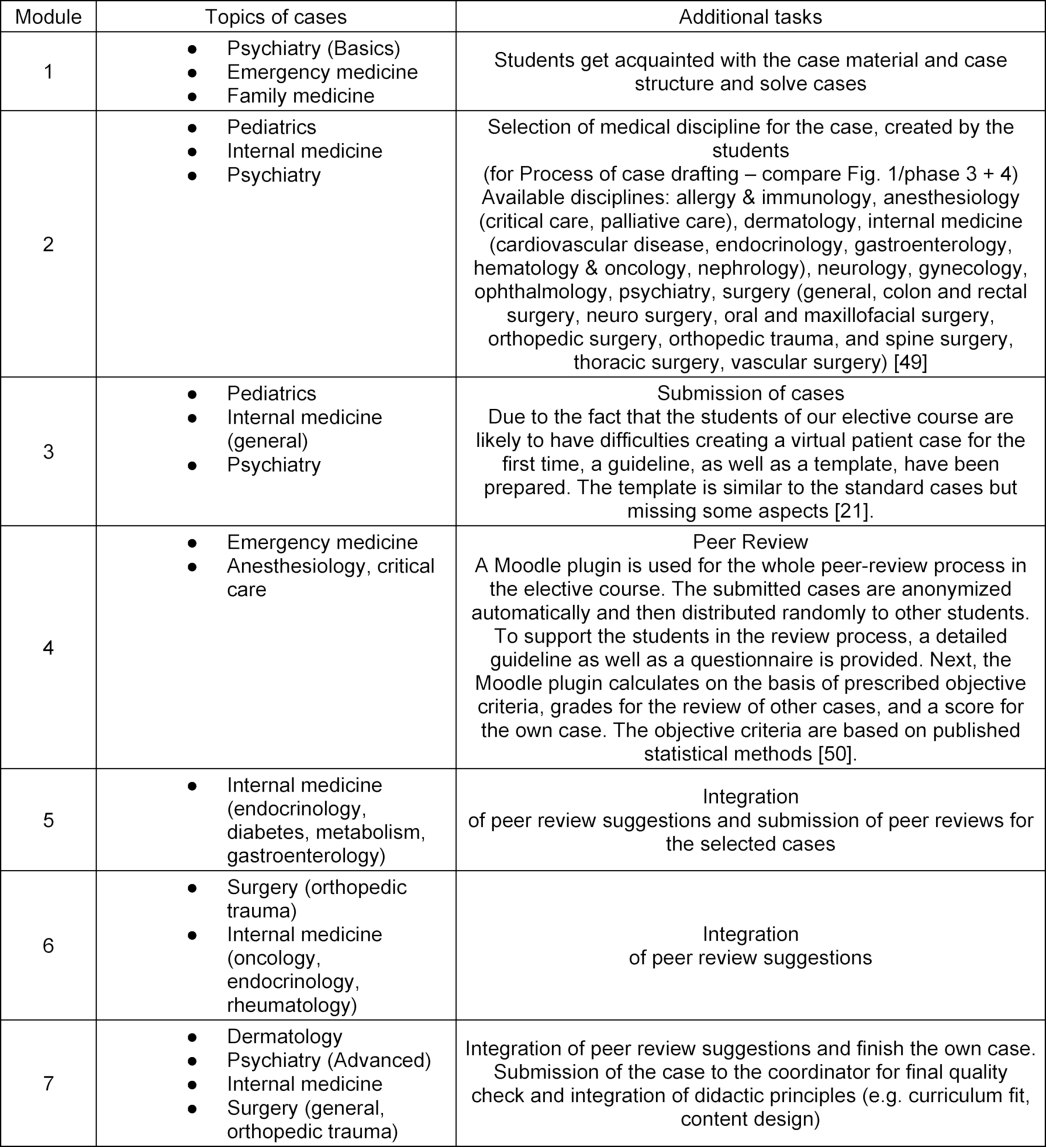
Thematic modules of the elective course

## References

[1] Tulving E, Thomson DM (1973). Encoding specificity and retrieval processes in episodic memory. Psychol Rev.

[2] Miller J, Bligh J, Stanley I, Al Shehri A (1998). Motivation and continuation of professional development. Br J Gen Pract.

[3] Swanwick T (2013). Understanding Medical Education: Evidence, Theory, and Practice.

[4] Coffield F, Moseley D, Hall E, Ecclestone K (2004). Learning styles and pedagogy in post-16 learning: a systematic and critical review.

[5] Mayer RE, Sims VK (1994). For whom is a picture worth a thousand words? Extensions of a dual-coding theory of multimedia learning. J Educ Psychol.

[6] Paivio A (1971). Imagery and Verbal Processes.

[7] Hounsell D, Entwistle N, Anderson C, Bromage A, Day K, Hounsell J, Land R, Litjens J, McCune V, Meyer E, Reimann N, Xu R (2005). Enhancing Teaching-Learning Environments in Undergraduate Courses Final Report to the Economic and Social Research Council.

[8] Ferguson E, James D, Madeley L (2002). Factors associated with success in medical school: Systematic review of the literature. Br Med J.

[9] Knowles MS (1980). The Modern Practice of Adult Education: From Pedagogy to Andragogy: Revised and Updates.

[10] Rooney JE (2003). Knowledge infusion. Assoc Manag.

[11] Ross B, Gage K, Bonk CJ, Grahahm CR, Corss J, Moore MG (2006). Global perspectives on blending learning. The Handbook of Blended Learning: Global Perspectives, Local Designs.

[12] Norberg A, Dziuban C, Moskal P (2011). A time-based blended learning model. Horizon.

[13] Teng YT, Bonk CJ, Kim KJ (2009). The trend of blended learning in Taiwan: perceptions of HRD practitioners and implications for emerging competencies. Hum Resour Dev Int.

[14] Graham CR, Bonk CJ, Grahahm CR, Corss J, Moore MG (2006). Blended learning systems: Definition, current trends, and future directions. The Handbook of Blended Learning: Global Perspectives, Local Designs.

[15] Garrison DR, Kanuka H (2004). Blended learning: Uncovering its transformative potential in higher education. Internet High Educ.

[16] Sharpe R, Benfield G, Roberts G, Francis R (2006). The undergraduate experience of blended e-learning: a review of UK literature and practice.

[17] Turk BR, Krexner R, Otto F, Wrba T, Löffler-Stastka H (2015). Not The Ghost in The Machine: Transforming Patient Data into E-Learning Cases Within A Case-Based Blended Learning Framework For Medical Education. Procedia - Soc Behav Sci.

[18] Garrison DR, Anderson T, Archer W (2010). The first decade of the community of inquiry framework: A retrospective. Internet High Educ.

[19] Garrison DR, Cleveland-Innes M, Bourne J, Moore J (2003). Critical factors in student satisfaction and success: Facilitating student role adjustment in online communities of inquiry. Elements of quality online education: into the mainstream.

[20] Seitz T, Gruber B, Preusche I, Loffler-Stastka H (2017). What causes the decrease in empathy among medical students during their university training?. Z Psychos Med Psychother.

[21] Turk B, Ertl S, Wong G, Wadowski PP, Löffler-Stastka H (2019). Does case-based blended-learning expedite the transfer of declarative knowledge to procedural knowledge in practice?. BMC Med Educ.

[22] Wadowski PP, Litschauer B, Seitz T, Ertl S, Löffler-Stastka H (2019). Case-based blended eLearning scenarios-adequate for competence development or more?. Neuropsychiatr.

[23] Williams B (2005). Case based learning - A review of the literature: Is there scope for this educational paradigm in prehospital education?. Emerg Med J.

[24] Bösner S, Pickert J, Stibane T (2015). Teaching differential diagnosis in primary care using an inverted classroom approach: Student satisfaction and gain in skills and knowledge. BMC Med Educ.

[25] Ertl S, Stastka L, Löffler-Stastka H (2020). Strukturiertes fallorientiertes Lernen. psychopraxis neuropraxis. Psychopraxis Neuropaxis.

[26] Med Universität Wien (2020). Studienziel & Qualifikationsprofil des Diplomstudiums Humanmedizin. Studium an der MedUni Wien.

[27] Ertl S (2020). Unpublished diploma thesis: Impact of e-learning on learning efficiency and interdisciplinary collaboration.

[28] Loosveld LM, Van Gerven PWM, Vanassche E, Driessen EW (2020). Mentors' Beliefs About Their Roles in Health Care Education: A Qualitative Study of Mentors' Personal Interpretative Framework. Acad Med.

[29] Löffler-Stastka H (2015). Do our medical students even want e-learning? A user rated evaluation of case based e-learning in undergraduate medical education at the medical university of Vienna. Adv Soc Sci Res J.

[30] Ertl S, Loeffler-Stastka H (2019). Case Based Blended Learning (CBBL) - a strategy to foster the transfer of declarative to procedural knowledge or more?.

[31] Ertl S, Loeffler-Stastka H (2018). Vienna tracking students in 25.000 exam results.

[32] Seitz T, Turk BR, Löffler-Stastka H (2015). Knowing the concern, concerned about not knowing-medical students' self-assessment of taking medical histories. Soc Sci Teach Res Adv Soc Behav Sci.

[33] Wadowski PP, Steinlechner B, Schiferer A, Löffler-Stastka H (2015). From clinical reasoning to effective clinical decision making-new training methods. Front Psychol.

[34] Löffler-Stastka H, Wong G (2020). Learning and competence development via clinical cases - what elements should be investigated to best train good medical doctors?. World J Meta-Analysis.

[35] Chéron M, Ademi M, Kraft F, Löffler-Stastka H (2016). Case-based learning and multiple choice questioning methods favored by students. BMC Med Educ.

[36] St George's University (2020). The Ultimate List of Medical Specialties.

[37] Koh GC, Khoo HE, Wong ML, Koh D (2008). The effects of problem-based learning during medical school on physician competency: A systematic review. CMAJ.

[38] Berkhof M, van Rijssen HJ, Schellart AJ, Anema JR, van der Beek AJ (2011). Effective training strategies for teaching communication skills to physicians: An overview of systematic reviews. Patient Educ Couns.

[39] Gartmeier M, Bauer J, Fischer MR, Hoppe-Seyler T, Karsten G, Kiessling C, Möller GE, Wiesbeck A, Prenzel M (2015). Fostering professional communication skills of future physicians and teachers: effects of e-learning with video cases and role-play. Instr Sci.

[40] Luo L, Cheng X, Wang S, Zhang J, Zhu W, Yang J, Liu P (2017). Blended learning with Moodle in medical statistics: An assessment of knowledge, attitudes and practices relating to e-learning. BMC Med Educ.

[41] de Araujo Guerra Grangeia T, de Jorge B, Franci D, Santos TM, Setubal MSV, Schweller M, de Carvalho-Filho MA Cognitive load and self-determination theories applied to e-learning.

[42] Antonoff MB, Verrier ED, Allen MS, Aloia L, Baker C, Fann JI, Iannettoni MD, Yang SC, Vaporciyn AA (2016). Impact of moodle-based online curriculum on thoracic surgery in-training examination scores. Ann Thorac Surg.

[43] Mandin H, Jones A, Woloschuk W, Harasym P (1997). Helping students learn to think like experts when solving clinical problems. Acad Med J Assoc Am Med Coll.

[44] Mandin H, Harasym P, Eagle C, Watanabe M (1995). Developing a" clinical presentation" curriculum at the University of Calgary. Acad Med.

[45] Kassirer JP (2010). Teaching clinical reasoning: Case-based and coached. Acad Med.

[46] Kopp V, Stark R, Fischer MR (2008). Fostering diagnostic knowledge through computer-supported, case-based worked examples: Effects of erroneous examples and feedback. Med Educ.

[47] Kiesewetter J, Ebersbach R, Görlitz A, Holzer M, Fischer MR, Schmidmaier R (2013). Cognitive problem solving patterns of medical students correlate with success in diagnostic case solutions. PLoS One.

[48] Khan BH (2005). Managing E-Learning: Design, Delivery, Implementation, and Evaluation.

[49] Löffler-Stastka H, Seitz T, Billeth S, Pastner B, Preusche I, Seidman C (2016). Significance of gender in the attitude towards doctor-patient communication in medical students and physicians. Wien Klin Wochenschr.

[50] TU Ilmenau (2017). Peer Assessment mit Moodle.

